# Differential effects of photobiomodulation interval schedules on brain cytochrome c-oxidase and proto-oncogene expression

**DOI:** 10.1117/1.NPh.7.4.045011

**Published:** 2020-12-08

**Authors:** Jorge L. Arias, Marta Mendez, Juan Ángel Martínez, Natalia Arias

**Affiliations:** a University of Oviedo, Neuroscience Laboratory, Department of Psychology, Oviedo, Spain; b INEUROPA, Instituto de Neurociencias del Principado de Asturias, Oviedo, Spain; c University of Oviedo, Escuela Politécnica de Gijón, Departamento Ingeniería Eléctrica, Electrónica, Computadores y Sistemas, Gijón, Spain; d Institute of Psychiatry, Psychology and Neuroscience, Maurice Wohl Clinical Neuroscience Institute, King´s College London, Department of Basic and Clinical Neuroscience, London, United Kingdom

**Keywords:** photobiomodulation, cytochrome c-oxidase, c-Fos, striatum, prefrontal cortex

## Abstract

**Significance:** Transcranial photobiomodulation (PBM) is a noninvasive neuromodulation technique capable of producing changes in the mitochondrial cytochrome c-oxidase (CCO) activity of neurons. Although the application of PBM in clinical practice and as a neurophysiological tool is increasing, less is known about how different treatment time intervals may result in different outcomes.

**Aim:** We evaluated the effects of different PBM treatment intervals on brain metabolic activity through the CCO and proto-oncogene expression (c-Fos).

**Approach:** We studied PBM effects on brain CCO and c-Fos expression in three groups of animals: Control (CN, n=8), long interval PBM treatment (LI, n=5), and short interval PBM treatment (SI, n=5).

**Results:** Increased CCO activity in the LI group, compared to the SI and CN groups, was found in the prefrontal cortices, dorsal and ventral striatum, and hippocampus. Regarding c-Fos expression, we found a significant increase in the SI group compared to LI and CN, whereas LI showed increased c-Fos expression compared to CN in the cingulate and infralimbic cortices.

**Conclusions:** We show the effectiveness of different PBM interval schedules in increasing brain metabolic activity or proto-oncogene expression.

## 1 Introduction

Transcranial photobiomodulation (PBM) is a noninvasive neuromodulation technique that has the ability to increase cellular metabolism and blood flow. It can be utilized for neuroprotection due to its role in reversing apoptotic signaling processes, and it has been found to promote synaptogenesis, among other actions.^1^ Although the precise cellular and molecular mechanisms underlying PBM are not yet fully understood, light in the 600- to 1200-nm wavelength range has significant PBMT (photobiomodulation therapy) capability.^2^

**Fig. 1:**
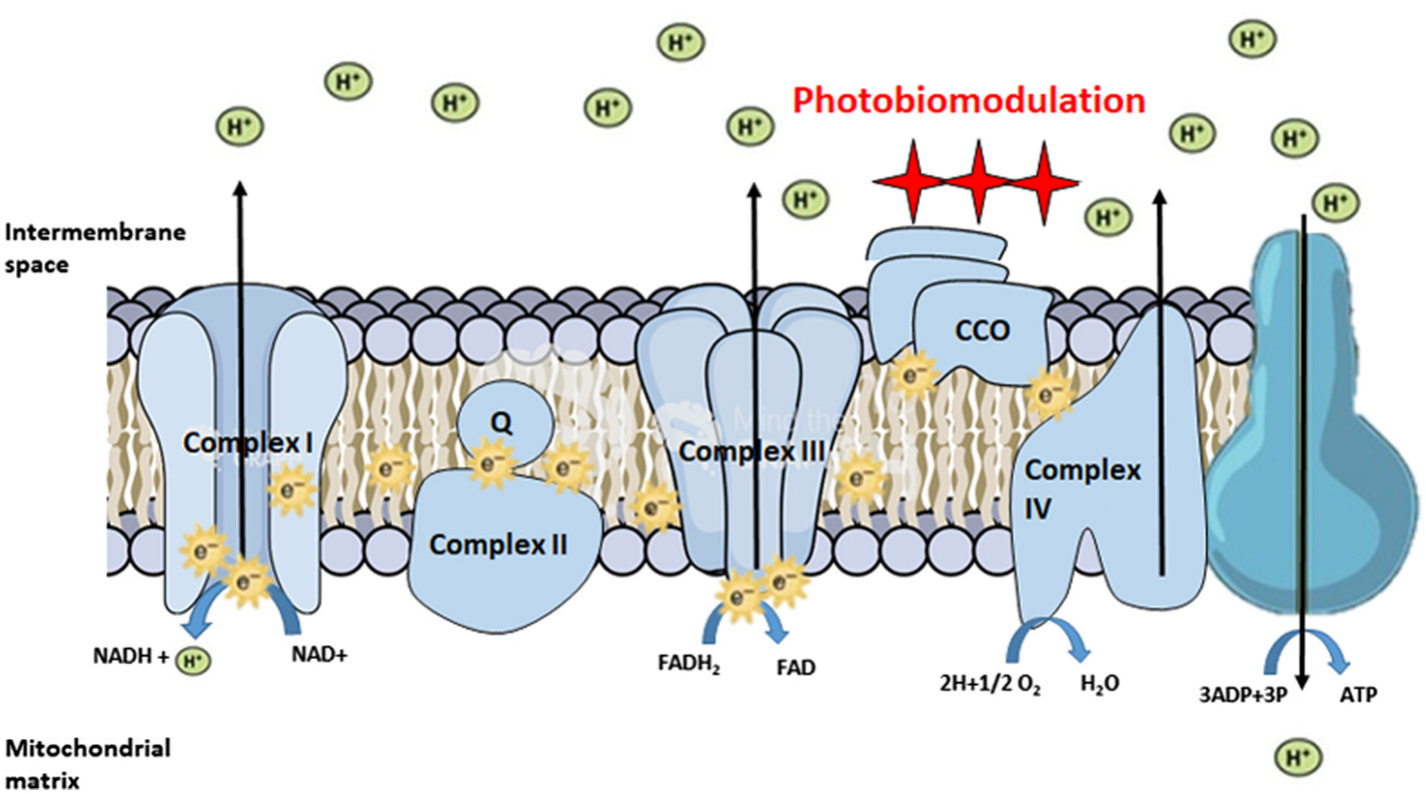
Mechanisms of action in PBMT strategies. PBM directly stimulates CCO (complex IV), facilitating its catalytic activity and inducing an increase in holoenzyme subunit assembly.

Transcranial PBMT is based on photon energy absorption and upregulation of cytochrome c-oxidase (CCO),^3^^,^^4^ which has resulted in neuroprotective effects in traumatic brain injury,^5^ ischemic stroke,^6^ Alzheimer’s disease,^7^ Parkinson’s disease,^8^ and psychological disorders such as depression and anxiety,^9^^,^^10^ as well as in age-related cognitive decline.^11^^,^^12^ Within PBMT, low-level light therapy is a new therapeutic technology that interacts with CCO inside the mitochondria, restoring electron transport chain activity,^13^^,^^14^ and therefore, improving energy metabolism (Fig. 1).

However, the effects on the brain of different time intervals between individual treatments have not been explored, although there is some evidence suggesting that this is an important parameter in other PBMT applications.^15^ Animal studies have demonstrated that a single PBMT administration is able to reduce cognitive and motor deficits in a traumatic brain injured (TBI) animal model.^16^^,^^17^ In this line, Cassano et al. (2015),^18^ using higher power (5 W) laser diodes for short intervals, were able to remit depressive symptoms in patients. Conversely, in humans with chronic or mild TBI, the only treatments that showed beneficial effects were long interval treatments performed three times a week for 6 weeks.^19^ Similarly, treatment for major depressive disorder is effective when administered twice a week for 8 weeks.^20^ Thus, the effects of different intervals (e.g., long versus short) of PBMT treatments on the brain have not been explored.

Another current debate in the field is whether pulsing or continuous light could have an effect of penetration depth and, hence, a differential effect on target activation, such as CCO. Studies by Henderson and Morries^2^ have shown that pulsed light yielded greater penetration through sheep skin, intact sheep heads, and living human tissue, but these results were not statistically significant and resulted in overall lower irradiance compared to continuous light. Conversely, on fixed human cadaver heads, there was no difference between pulse- and continuous-wave laser light.^21^ However, in animal TBI studies, pulse-wave laser light was superior to continuous-wave,^22^ and it was found to avoid unpleasant heat damage due to continuous irradiation periods.^23^^,^^24^

Our aim was to evaluate the effect of different intervals (long versus short) of PBMT on neural activity through c-Fos immunohistochemistry and CCO histochemistry. It was recently shown that PBM affects the production of reactive oxidative species and increases intracellular ${\mathrm{Ca}}^{2+}$,^25^ which can initiate the Ras/extracellular signal-regulated kinase (ERK) cascade,^26^ producing long-lasting effects on cells and suggesting the potential expression of some proto-oncogenes such as c-Fos. However, the effect of PBM on proto-oncogene expression has not been explored.

The intracellular transcription of c-Fos will need an adenosine triphosphate (ATP) source, and so we wanted to explore energy metabolism through the study of CCO activity. For this purpose, we measured CCO activity through optical densitometry. It is well known that almost all the energy obtained in neurons is derived from oxidative phosphorylation in the mitochondria, and CCO is one of the key energy generating enzymes in this process. The PBM action mechanism is based on photon energy absorption and upregulation of cytochrome oxidase,^3^^,^^4^ which has resulted in behavioral and metabolic neuroprotection in animal models of retinal neurotoxicity,^27^^,^^28^ traumatic brain injury,^29^ and autoimmune encephalomyelitis.^30^ We hypothesized that PBMT would increase neuronal respiration and boost brain energy metabolic capacity,^31^ which could be reflected in an increase in brain regional CCO activity.

## 2 Materials and Methods

### 2.1 Subjects

We used 18 male Wistar rats (290 to 330 g). They were maintained under standard laboratory conditions (20°C to 22°C, 65% to 70% relative humidity, and a 12-h light/dark cycle). All procedures were carried out according to the European Parliament and the Council of the European Union 2010/63/UE and approved by the Oviedo University committee for animal studies. The animals were randomly distributed into three groups, one control and two differential interventions: control group (CN, n=8), long interval PBMT group (LI, n=5), and short interval PBMT group (SI, n=5).

### 2.2 Photobiomodulation Therapy

Animals in the long interval PBMT group (LI) received 1 session of low-light level treatment a day for 7 days (between 9:00 and 10:00 a.m.). Animals in the short interval PBMT group (SI) received four PBMT sessions for a total of 3 min within 30 h.^32^ The administration of the light in the SI group took place at 10:00 a.m., 17:30 p.m., 01:00 a.m., and 8:30 a.m. PBMT was delivered by a 670±10-nm wavelength LED array (Quantum Devices Warp 10, Barneveld), the device (5.1 cm diameter, 1073 mW, $50\;{\mathrm{mW}/\mathrm{cm}}^{2}$) delivered for 3 min a total of ${9\;J/\mathrm{cm}}^{2}$. The device was placed on the midline of the dorsal surface of the animal’s shaved head in the region between the eyes and ears. To avoid PBMT to other brain regions, an opaque material covered the rest of the device; moreover, in order to assure consistency, the same researcher delivered the treatment in all groups and conditions. The equipment used to measure the power of the light source was a PM160 Optical Power Meter by THORLABS (New Jersey). Animals in the control group (CN) were subjected to the same protocol, except that the LED device was turned off.

### 2.3 Brain Processing

All the animals were decapitated 90 min after the last PBMT exposure. Then, the brains were removed intact, frozen rapidly in isopentane (Sigma-Aldrich, Germany), and stored at −40°C. We use 90-min postprobe because the cellular c-fos proto-oncogene is an immediate early expression gene whose induction is one of the first cellular responses after the application of a variety of stimuli. This induction is rapid and transient and can encode its protein with a peak in expression between 1 and 2 h.^33^^,^^34^

Coronal sections (30  μm) of the brain were cut at −20°C in a cryostat (Leica CM1900, Germany). Distance in mm of brain regions counted from bregma was: +3.20  mm for anterior cortices, including the infralimbic (IF), prelimbic (PL), and cingulate (CG) cortex; +1.2  mm for the dorsal striatum (STR), including the anterodorsal, anterolateral, and anteromedial; +1.2  mm for the accumbens core (ACC) and accumbens shell (ACS); and −3.3  mm for the cornu ammonis (CA)1, CA3, and dentate gyrus (DG) subareas of the dorsal hippocampus. The selected brains regions were anatomically defined according to the atlas by Paxinos and Watson.^35^

#### 2.3.1 Cytochrome c-oxidase histochemistry

The protocol used was the same one previously described.^36^ Briefly, sets of tissue homogenate standards from Wistar rat brains were included with each bath of slides. Sections and standards were incubated in 0.1 phosphate buffer (PB) with 10% (w/v) sucrose and 0.5 (v/v) glutaraldehyde. Then, baths of 0.1M PB with sucrose were given. Subsequently, 0.05M Tris buffer was applied. Then, sections and standards were incubated in a solution with 0.0075% cytochrome-c (w/v), 0.002% catalase (w/v), 5% sucrose (w/v), 0.25% dimethylsulfoxide (v/v), and 0.05% diaminobenzidine tetrahydrochloride in 0.1M PB. The reaction was stopped by fixing the tissue in buffered 4% (v/v) formalin. Finally, the slides were dehydrated and cleared with xylene. CCO histochemical staining intensity was quantified by densitometric analysis using a computer-assisted image analysis workstation (MCID, Interfocus Imaging Ltd., Linton, England). A total of 12 measurements were taken per brain region. These measures were averaged to obtain one mean per region for each animal, and they were expressed as arbitrary units of optical density (OD).

#### 2.3.2 c-Fos activity

Five animals from each group were processed immunocytochemically for c-Fos. The sections were mounted on gelatinized slides. Then, the sections were post-fixed in buffered 4% paraformaldehyde (0.1M, pH 7.4) for 30 min and rinsed in phosphate-buffered saline (PBS) (0.01 M, pH 7.4). They were subsequently incubated for 15 min with 3% hydrogen peroxidase in PBS to remove endogenous peroxidase activity and then washed twice in PBS. After blocking with PBS solution containing 10% Triton X-100 (PBS-T) (Sigma, USA) and 3% bovine serum albumin for 30 min, sections were incubated with a rabbit polyclonal anti-c-Fos solution (1:10.000) (Santa Cruz Biotech, sc-52, USA) diluted in PBS-T for 24 h at 4°C in a humid chamber. Slides were then washed three times with PBS and incubated in a goat anti-rabbit biotinylated IgG secondary antibody (Pierce, USA; diluted 1:200 in incubating solution) for 2 h at room temperature. They were washed three times in PBS and reacted with avidin–biotin peroxidase complex (Vectastain ABC Ultrasensitive Elite Kit, Pierce) for 1 h. After two washes in PBS, the reaction was visualized, treating the sections for about 3 min in a commercial nickel–cobalt intensified diaminobenzidine kit (Pierce). The reaction was finalized by washing the sections twice in PBS. Slides were then dehydrated through a series of graded alcohols, cleared with xylene, and cover-slipped with Entellan (Merck, USA) for microscopic observation. All the immunocytochemistry procedures included sections that served as controls where the primary antibody was not added. Slides containing sections of a specific brain region were stained at the same time. Slides were coded so that the investigator who performed the entire analysis would have no knowledge of the group to which the individual subjects belonged.

The total number of c-Fos positive nuclei was quantified in three alternate sections 30  μm apart. Quantification was done by systematically sampling each of the regions selected, using a microscope (Olympus BH-2, Japan) attached to an analog camera (Sony XC-77, Japan) and a TV monitor (300×total magnification). c-Fos-positive nuclei were defined based on homogeneous gray-black stained elements with a well-defined border. Finally, the mean count for three sections was calculated for each subject and region ($\text{number of positive nuclei}/150\;{\mu m}^{2}$).

### 2.4 Statistical Analysis

All data were analyzed by the Sigma-Stat 3.2 program (Systat, Richmond) and expressed as the mean±SEM. The results were considered statistically significant if p<0.05. A one-way repeated measures of analysis of variance (ANOVA) was used for the statistical comparison of the CCO activity and c-Fos expression values between the groups. Post-hoc multiple comparison analyses were carried out when possible, using pairwise Tukey tests. Moreover, a nonparametric Kruskall–Wallis test (H) for independent samples was performed when normality or equal group variances failed.

## 3 Results

### 3.1 CCO Activity

**Fig. 2:**
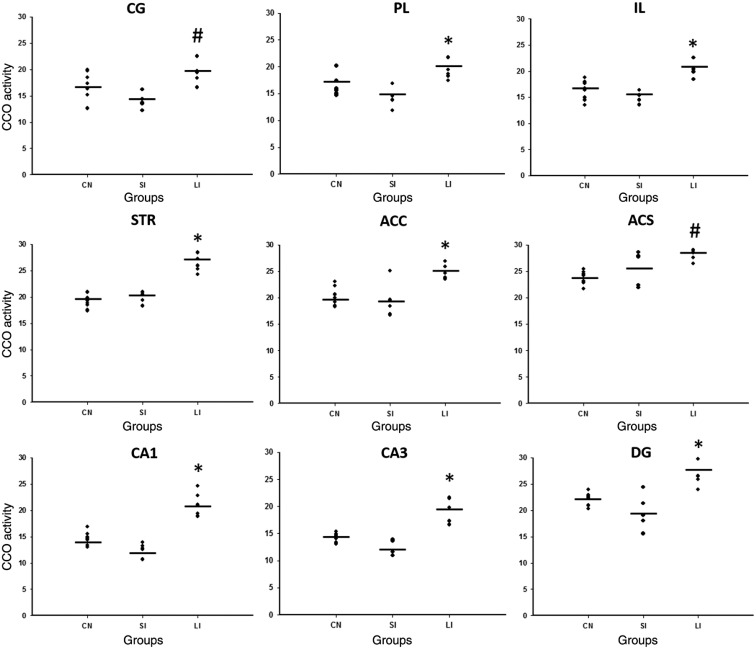
CCO histochemistry in the sampled regions where significant differences were found in cingulate (CG), prelimbic (PL) and infralimbic (IL) cortices, dorsal striatum (STR), ACC, ACS, CA1, CA3, and dentate gyrus (DG) subregions of the hippocampus. *p<0.05 versus SI and CN groups. Lines represent mean.

When we explored the CCO brain activity, we found higher activity in the LI group compared to the other groups in PL (F2,17 = 9.699, p=0.002) between SI (p=0.002) and CN (p=0.028), IL (F2,17 = 15.980, p<0.001) compared to SI (p<0.001) and CN (p=0.002), STR (F2,17 = 49.566, p<0.001) compared to SI (p<0.001) and CN (p<0.001), ACC (F2,17 = 9.335, p=0.002) compared to SI (p=0.003) and CN (p=0.007), CA1 (F2,17 = 41.314, p<0.001) compared to SI (p<0.001) and CN (p<0.001), CA3 (F2,17 = 27.543, p<0.001) compared to SI (p<0.001) and CN (p<0.001), and DG (F2,17 = 12.407, p<0.001) compared to SI (p<0.001) and CN (p=0.005). Moreover, increased CCO activity in CG (F2,17 = 7.750, p=0.005) was found between LI compared to the SI group (p=0.004) (Fig. 2).

### 3.2 c-Fos Activity

**Fig. 3:**
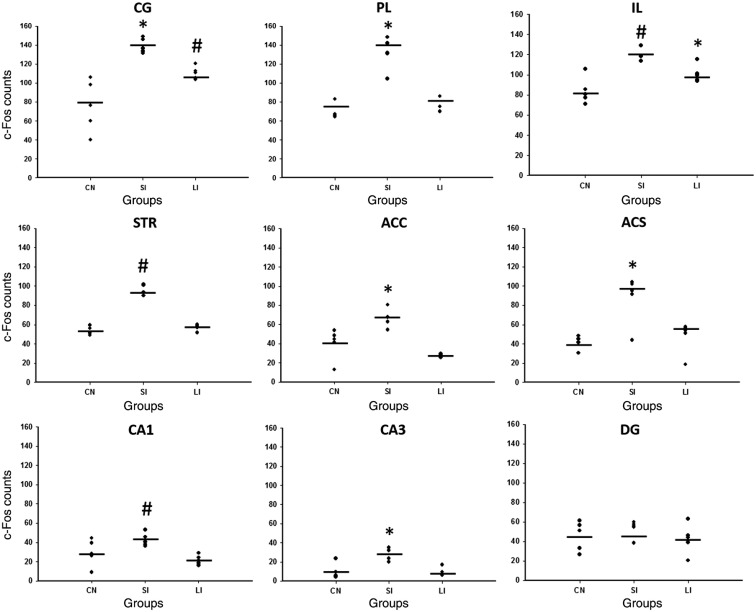
c-Fos immunoreactivity expression in the sampled regions where significant differences were found in cingulate (CG), prelimbic (PL) and infralimbic (IL) cortices, dorsal striatum (STR), ACC, ACS, CA1, CA3 subregions of the hippocampus. *p<0.05 versus SI and CN groups. #p<0.05 versus. CN group. No differences were found in the dentate gyrus (DG). Lines represent mean.

We found a significant increase in proto-oncogene expression, c-Fos, in the SI group compared to LI and CN. One-way ANOVA showed significant differences in PL (F2,14 = 46.233, p<0.001) compared to LI (p<0.001) and CN (p<0.001), IL (F2,14 = 16.944, p<0.001) compared to LI (p=0.026) and CN (p<0.001), CG (F2,14 = 17.912, p<0.001) compared to LI (p=0.046) and CN (p<0.001), STR (H2 = 10.220, p=0.006) compared to CN (p<0.05), ACC (F2,14 = 17.350, p<0.001) compared to LI (p<0.001) and CN (p=0.006), ACS (F2,14 = 9.717, p=0.003) compared to LI (p=0.011) and CN (p=0.004), and CA3 (F2,14 = 12.185, p=0.001) compared to LI (p=0.002) and CN (p=0.004). Moreover, differences were found between LI and CN in IL (p=0.042) and CG (p=0.017). Furthermore, increased c-Fos expression was found in the SI group in comparison with LI (p=0.011) in CA1 (F2,14 = 6.401, p=0.013). Finally, no differences between the groups were found in DG (F2,14 = 0.861, p=0.447) (Fig. 3).

## 4 Discussion

Transcranial PBM involves exposing cells or tissues to low level light photons in the wavelength range from red to near-infrared light (600 to 1200 nm).^37^ PBM is an emerging therapeutic technology that interacts with CCO inside the mitochondria, restoring the electron transport chain activity,^13^^,^^14^ and therefore, improving energy metabolism. Transcranial PBM is based on photon energy absorption and upregulation of CCO,^3^^,^^4^ which improves neuronal metabolic capacity in the rat brain,^31^ increased ATP content,^38^ and altered mitochondrial dynamics.^39^ Moreover, transcranial PBM has produced neuroprotective effects in several disorders, such as traumatic brain injury,^5^ ischemic stroke,^6^ Alzheimer’s disease,^7^ Parkinson’s disease,^8^ and anxiety and depression.^9^^,^^10^ This treatment also shows promising results in improving cognitive decline related to aging.^11^^,^^12^ However, despite its promising role as a therapeutic agent, less is known about the impact of different intervals of PBMT treatment on the brain function.

Our results demonstrated that PBMT was able to boost brain metabolism not only by increasing mitochondrial CCO activity but also through the activation of proto-oncogene expression. Moreover, we found a differential brain effect depending on the intervals between successive stimulations (short versus long intervals).

Indeed, the LI group showed increased CCO activity in all the hippocampal subregions, infralimbic and prelimbic cortices, striatum, and accumbens nucleus, compared to the SI and CN groups. These results could be explained by the biological photoacceptor nature of CCO.^28^^,^^40^ Specifically, absorption of the photons delivered in PBM seems to promote an increase in the availability of electrons for the reduction of molecular oxygen in the catalytic center of CCO, thus increasing the mitochondrial membrane potential, ATP levels, and reactive oxygen species, which leads to increased mitochondrial function.^41^

Furthermore, it has been shown that PBM is able to photodissociate NO from CCO,^42^ which could reverse the mitochondrial inhibition of cellular respiration that exists as a result of excessive NO binding.^43^ Thus, NO would no longer compete with oxygen in binding to the catalytic metal centers of CCO, allowing for an influx of oxygen. Therefore, enzymatic activity and respiration could return to baseline, increasing CCO activity and ATP production, which is in line with our findings.

Another interesting finding is the general activation found in the brain regions studied. The relationship between the hippocampus and other brain regions in supporting memory processes is well known. Regarding this, the cooperation between the hippocampus and the striatum has been reported during episodic encoding.^44^^,^^45^ The episodic memory network comprises the cingulate cortex^46^^,^^47^ and medial prefrontal cortex,^48^ as well as the aforementioned regions, highlighting that they belong to an established network. Indeed, it has been suggested that when transcranial light irradiation coincides geographically with the stimulation of some brain regions involved in any intrinsic network, a therapeutic benefit will be extended to the entire brain network.^49^ In this line, a systematic effect of low-PBMT has been previously described by Naeser et al.^50^ where benefit to cerebral perfusion was observed when PBMT was applied to an acupuncture point on the foot. These systematic attributes to PBMT have been supported by changes in nitric oxide levels^51^ and excitotoxic modulation secondary to brain injury.^29^ Moreover, although there is a lack of studies that have tested whether neurotransmission is involved in mediating the protective effects of PBM, it has been demonstrated that PBMT increases endothelial nitric oxide synthase activity^52^ leading to a transient increase in NO, which has an important role in neurotransmission and signal transduction.^53^^,^^54^ So, our CCO results could be supporting this systematic effect where activation of some of the elements of the network could trigger the activation of deeper brain regions involved, as it has been demonstrated when PBMT was applied to the default mode network.^55^^,^^56^

In fact, this dose fractionation based on long intervals was recently found to produce cognitive improvement, evaluated with the minimental state exam, in mild to moderately severe dementia patients and patients with Alzheimer’s disease.^57^ In addition, research has also shown that PBMT fractionation protocols that include prophylactic doses given before neurotoxic metabolic lesions are also effective in preventing neurodegeneration.^58^

Less explored has been the effect of PBMT on proto-oncogene expression, such as c-fos. Our results show that c-Fos expression increased after SI intervention in the CA1 and CA3 areas of the hippocampus, prefrontal cortices, striatum, and accumbens nucleus. c-Fos expression reflects evoked cellular activation following stimuli application,^59^ such as, in our case, PBMT administration. We hypothesized that this increased c-Fos expression could be explained by two complementary mechanisms. First, the ability of low-level light to activate mitogen-activated protein kinases (MAPK) phosphorylation initiates the ERK cascade until activating c-Fos expression.^60^ Second, it was recently demonstrated that low-light level therapy could contribute to increased intracellular ${\mathrm{Ca}}^{2+}$, which is a versatile second messenger^61^^,^^62^ involved in transcriptional regulation via protein kinase A (PKA), MAPK, and calmodulin-stimulated protein kinase.^63^ Moreover, an increase in ${\mathrm{Ca}}^{2+}$ intracellular concentration can initiate the Ras/ERK cascade,^27^ followed by increased c-Fos expression. Finally, PBM has been found to trigger retrograde mitochondrial signaling.^64^ This refers to signals and communications passing from the mitochondria to the nucleus of a cell, rather than vice versa. Thus, our findings might suggest that longer administration intervals could be driving the aforementioned communication between mitochondrial changes and the nucleus.^65^ Moreover, it is important to note that a differential increase in c-Fos expression could be appreciated among brain regions where prefrontal cortices showed the higher rates compared to subcortical regions such as striatum, hippocampus, and accumbens nucleus. These results are in line with Ref. 22, who demonstrated that near-infrared light penetrated the brain and scattered, thus establishing a gradient of light energy as the photons were absorbed and distributed within the brain tissue. So, these results could be supporting the light fluence gradient within the brain.

Finally, although our study with continuous light at different schedules proved different brain effects, other studies have proved that pulsing PBMT was able to change the spiking in parvalbumin n-positive interneurons and reduced the levels of amyloid β peptides in the brain of Alzheimer’s mice model.^66^ Moreover, Henderson and Morries^51^^,^^67^ studied the relevance of changes in power densities to achieve sufficient light penetration to the brain. All these results pointed out that future research should focus on how varying treatment parameters can change brain function, and how treatments can potentially be tuned for differing brain ailments.

In conclusion, our results add experimental evidence about the differential effects of PBMT intervals on brain stimulation. LI schedules have been shown to increase CCO activity in many structures that take part in the limbic memory network, whereas SI intervention enables c-Fos expression. This study may facilitate the development of new strategies to boost cortical and subcortical neurocognitive activity. Further studies should be carried out to explore PBMT systemic administration effects following these intervals on the same molecular mechanisms within the neurons.

## Code, Data, and Materials Availability

The authors have declared that the manuscript does not have associated code. Moreover, the authors have declared the availability of data used in the research results reported in the manuscript and have provided specific access information or restrictions (i.e., persistent weblinks to repository access addresses). Finally, the authors have declared the availability of materials, explained any restrictions on materials (i.e., materials transfer agreements), or provided supplementary materials for the purpose of reproducibility.
